# Correction: Identifcation, expression, and purifcation of DNA cytosine 5-methyltransferases with short recognition sequences

**DOI:** 10.1186/s12896-022-00770-6

**Published:** 2022-12-01

**Authors:** Fumihito Miura, Miki Miura, Yukiko Shibata, Yoshikazu Furuta, Keisuke Miyamura, Yuki Ino, Asmaa M. A. Bayoumi, Utako Oba, Takashi Ito

**Affiliations:** 1 Department of Biochemistry, Kyushu University Graduate School of Medical Sciences, 3-1-1 Maidashi, Higashi-Ku, Fukuoka, 812-8582 Japan; 2 Division of Infection and Immunity, Research Center for Zoonosis Control, Hokkaido University, Sapporo, 001-0020 Japan; 3 Department of Biochemistry, Faculty of Pharmacy, Minia University, El-Minia, 61511 Egypt; 4 Department of Pediatrics, Kyushu University Graduate School of Medical Sciences, 3-1-1 Maidashi, Higashi-Ku, Fukuoka, 812-8582 Japan

**Correction to: BMC Biotechnol (2022) 22:33**

 10.1186/s12896-022-00765-3

Following publication of the original article [1], the authors informed us that the Funding section needs to be updated due to the lack of prefix “JP” to three grant numbers.

Incorrect 22ama121022j0001 → Correct JP22ama121022j0001

Incorrect 17H06305 → Correct JP17H06305

Incorrect 20H03243 → Correct JP20H03243

The original article has been corrected.

## References

[1] Miura (2022). BMC Biotechnol.

